# Elderly Male With Epstein-Barr Virus (EBV)-Induced Pure Red Cell Aplasia

**DOI:** 10.7759/cureus.61970

**Published:** 2024-06-08

**Authors:** Benjamin J McCormick, Ahmad Ghorab, Sammer M Elwasila, Marwan Shaikh

**Affiliations:** 1 Internal Medicine, Mayo Clinic, Jacksonville, USA; 2 Hematology and Oncology, Mayo Clinic, Jacksonville, USA; 3 Infectious Disease, Mayo Clinic Alix School of Medicine, Jacksonville, USA

**Keywords:** ebv-induced prca, anemia, non-malignant hematology, epstein barr virus (ebv), pure red cell aplasia (prca)

## Abstract

Pure red cell aplasia (PRCA) is a rare hematologic disorder presenting with symptomatic normocytic anemia with preservation of other bone marrow cell lineages that may be acquired in adulthood due to malignancy, autoimmune disease, and infections. PRCA has been attributed to Epstein-Barr virus (EBV) in patients with underlying malignancy; however, we present a rare case of EBV-related PRCA in a previously healthy elderly male without an underlying malignancy who developed transfusion-dependent anemia that responded to glucocorticoids, rituximab, and intravenous immunoglobulins.

## Introduction

Pure red cell aplasia (PRCA) is a rare hematologic disorder characterized by normocytic anemia, reticulocytopenia, and absence of erythroid precursors in the bone marrow with preservation of other marrow cell lineages [1]. Clinical presentation includes symptomatic effects of anemia, such as malaise and dyspnea. PRCA can be classified into Diamond-Blackfan anemia (DBA) and acquired PRCA. Acquired PRCA may be idiopathic or secondary to thymoma, malignancy, infection (especially parvovirus), autoimmune disease, medication/toxin exposure, or ABO-mismatched hematopoietic stem cell transplantation [1]. It is exceedingly rare for adults to develop Epstein-Barr virus (EBV)-related PRCA without underlying malignancy. We report a case of EBV-related PRCA in a previously healthy elderly male without underlying malignant contribution.

## Case presentation

**Figure 1:**
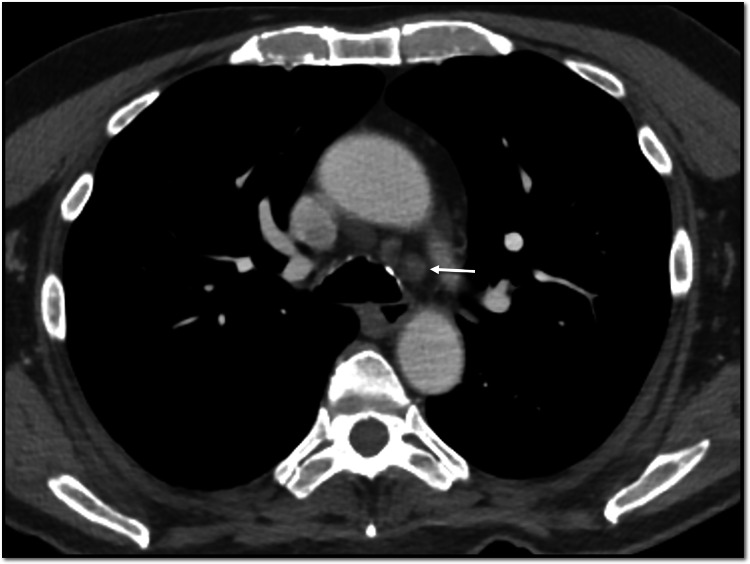
CT chest reveals mediastinal lymphadenopathy Arrow indicates low left paratracheal nodal enlargement, max 1 cm.

**Figure 2:**
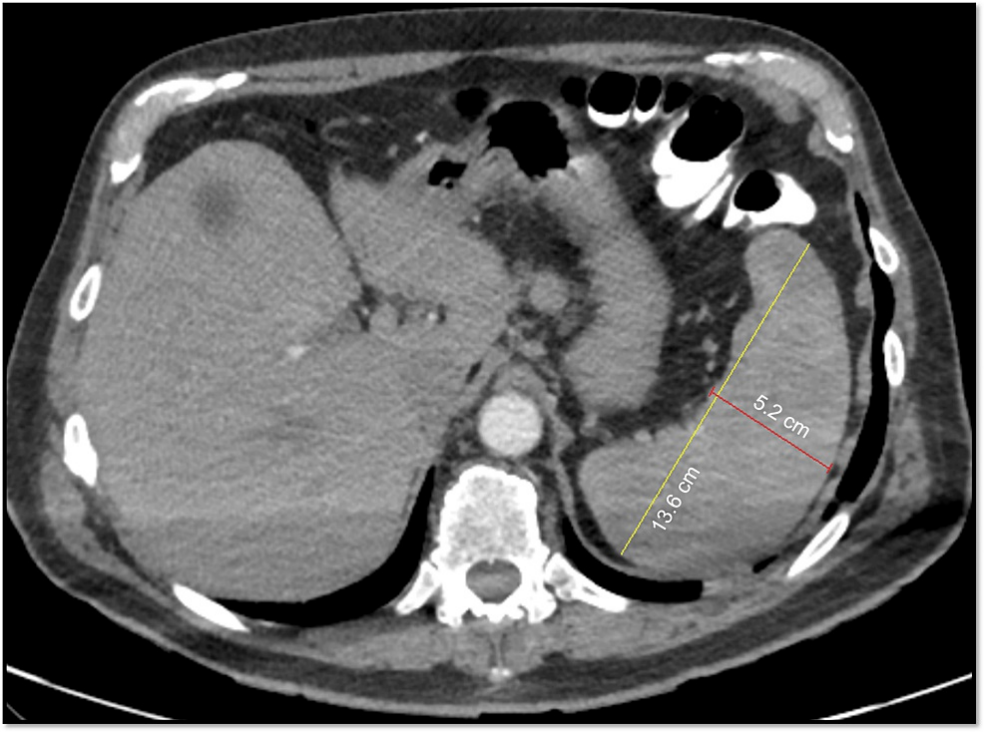
CT abdomen revealing splenomegaly (13.6 cm x 5.2 cm)

A 74-year-old male from Latin America with a prior history of hypertension and dyslipidemia developed unilateral cervical lymphadenopathy, fever, malaise, and diffuse erythematous rash of all extremities while traveling in South America at which time his complete blood count (CBC) was normal, including hemoglobin 14 g/dL. He received a five-day course of oral glucocorticoids. Two weeks after returning to Latin America, he developed severe malaise, generalized weakness, and intermittent fever with resolution of the rash and lymphadenopathy. Hemoglobin was 4.5 g/dL (normal range: 11.6-15.0 g/dL) with reticulocytes 1.5x10^9/L (normal range: 23-100x10^9/L). Bone marrow biopsy revealed erythroid aplasia and 2+ reticulin fibrosis without blasts or ringed sideroblasts and a normal blast count. Bone marrow aspirate was inadequate for evaluation of cellularity due to a lack of particles. Flow cytometry showed a normal karyotype. No B-cell or T-cell receptor gene rearrangements were seen. Bone marrow next-generation sequencing revealed DNMT3A/U2AF1 mutations (40.5% and 39.8% allele frequency, respectively) with negative JAK and HFE testing. Computed tomography (CT) chest with intravenous contrast showed mediastinal lymphadenopathy (maximum 1.0 cm) (Figure 1). CT abdomen with intravenous and oral contrast showed splenomegaly (13.6 cm)(Figure 2). He required 3-5 units of packed red blood cells weekly to maintain hemoglobin greater than 7 mg/dL. He received four days of intravenous methylprednisolone (40 mg daily) followed by a three-week prednisone taper. He subsequently received IVIG and three doses of rituximab, intravenous immunoglobulins. Finally, he received five days of cyclosporine, although this was discontinued due to inability to monitor serum cyclosporine levels near his home. Despite these treatment, he had no improvement in anemia. He was transferred to our institution for further evaluation and treatment two months after initial symptoms.

Upon arrival in the United States, he complained primarily of malaise and denied unintentional weight loss, night sweats, or chills. His vital signs were within normal limits except sinus tachycardia. The physical exam revealed conjunctival pallor without jaundice or splenomegaly.

**Figure 3:**
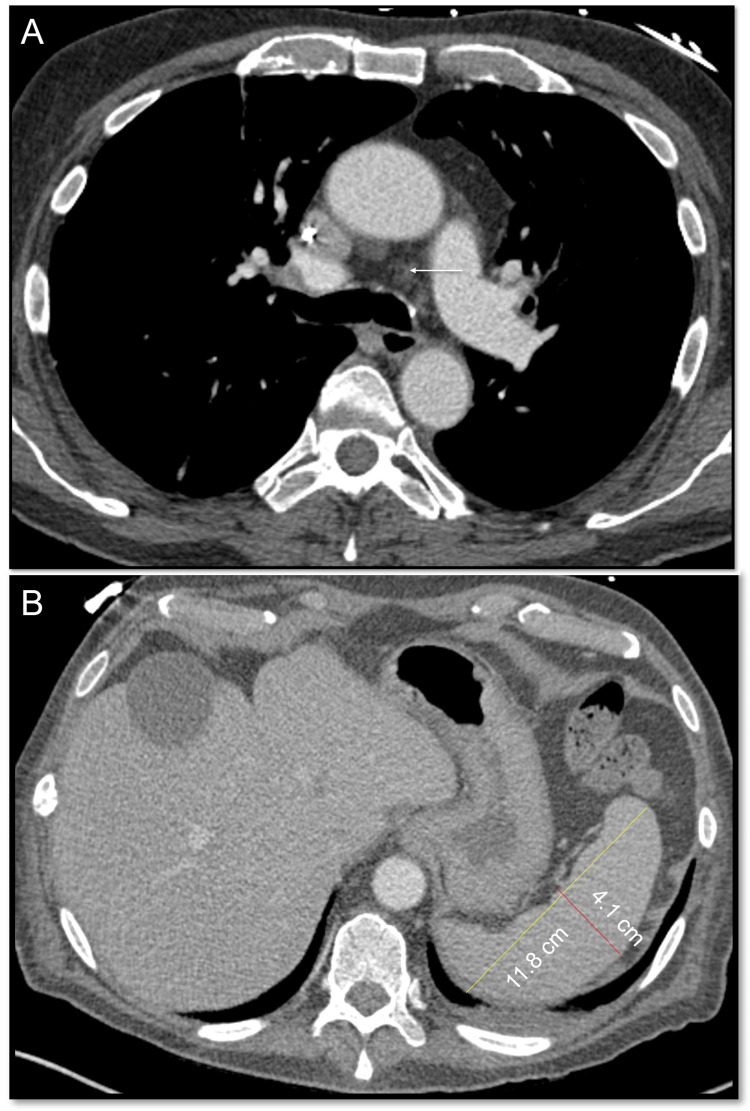
Repeat CT chest (A) Repeat CT chest shows resolution of lymphadenopathy (arrow); (B) repeat CT abdomen shows resolution of splenomegaly.

Laboratory findings with normal ranges listed parenthetically included hemoglobin, 6.1 g/dL (13.2-16.6 g/dL); mean corpuscular volume, 92 fL; platelets, 111x10^9/L (135-317x10^9/L); white blood cells, 3x10^9/L (3.4-9.6x10^9/L) without atypia; absolute reticulocytes, 54x10^9/L (30.4-110.9x10^9/L); haptoglobin, < 14 mg/dL (30-200 mg/dL); lactate dehydrogenase, 454 IU/L (122-222 IU/L), C-reactive protein, 27.9 mg/L (0-3 mg/L); ferritin, 1,718 mcg/L (24-336 mcg/L); erythropoietin, 86.1 mIU/mL (2.6-18.5 mIU/mL); and fibrinogen, 498 mg/dL (200-400 mg/dL). Antinuclear antibody and paroxysmal nocturnal hemoglobinuria tests were negative. There were no nutritional deficiencies. A peripheral smear was unremarkable. EBV serologies revealed IgM-/IgG+ with 4,630 copies/mL via polymerase chain reaction (PCR) testing. Other viral serologies, including cytomegalovirus, and tuberculosis testing were negative. Direct and indirect Coombs' tests were positive (IgG/C3+), including anti-E IgG antibodies. Urinalysis showed 1+ urobilinogen but was negative for blood or erythrocytes. A repeat CT of the chest and abdomen revealed no splenomegaly or lymphadenopathy (Figures 3A, 3B).

The patient received one dose of rituximab on day two of hospitalization and two days of prednisone (60 mg daily) with increasing transfusion independence. He received only one blood transfusion for hemoglobin less than 7 mg/dL throughout his seven-day hospitalization (partially limited by diffuse antibody presence narrowing available units). He was discharged home to Latin America on day seven of hospitalization with planned outpatient lab monitoring and virtual hematology clinic follow-up. At the time of discharge, the patient’s hemoglobin was 8.2 g/dL, absolute reticulocytes 93x10^9/L, white blood cell count 5.4x10^9/L, and platelet count 370x10^9/L. Unfortunately, the patient passed away due to an unrelated respiratory disease about two months after discharging home to Latin America.

## Discussion

We present a case of an elderly male without contributory medical history who developed EBV infection followed weeks later by new-onset acquired pure red cell aplasia. In a recent study of 22 adult patients with PRCA in India, etiologies were attributed to thymoma (4), tuberculosis (2), medications (2), hematologic malignancy (2), and idiopathic (10) [2]. A study in China demonstrated seven cases of PRCA attributed to cytomegalovirus or EBV in children less than 36 months old [3]. Multiple cases have been reported involving latent EBV-related PRCA in adult patients with underlying relapsed hematologic malignancy [2,4]. The pathophysiology of EBV-related PRCA is not fully understood; however, one common theory is that the prominent T-cell suppressor response meant to inhibit the polyclonal B-cell activation by EBV results in T-cell-mediated erythropoietin suppression [1,5]. However, it is exceedingly rare for adults to develop EBV-related PRCA without underlying malignancy, including a single case reported in a 19-year-old patient in 1984 [5].

Our workup revealed latent EBV infection that developed at least two weeks prior to testing, temporally consistent with the clinical syndrome seen two months prior. Positive direct and indirect Coombs' tests with diffuse antibodies were consistent with a warm autoimmune hemolytic anemia, which could be related to EBV, IVIG, or also immunization from partially incompatible blood transfusions. PRCA diagnosis was based on bone marrow biopsy showing erythroid aplasia with reticulocytopenia associated with transfusion-dependent normocytic anemia. Cytogenetics revealed DNTM3A/U2AF1 mutations, which are seen in myelodysplastic syndromes, acute myeloid leukemia, and myelofibrosis; however, hematopathology via bone marrow biopsy revealed no evidence of these malignancies. Reticulin fibrosis was present; therefore, he could have had subclinical myelofibrosis given otherwise normal hematopoiesis. Without JAK mutations, he did not meet World Health Organization criteria for primary myelofibrosis with a known cause of reactive fibrosis in EBV infection [6]. Although exceedingly rare in adults without malignancy, the epidemiological temporality and reversibility of this case were consistent with EBV-induced PRCA.

## Conclusions

Pure red cell aplasia is a rare hematologic disorder presenting with symptomatic normocytic anemia with preservation of other bone marrow cell lineages that may be acquired in adulthood. Acquired PRCA has been associated with EBV infection, especially in adults with underlying malignancy. We report a case of EBV-related PRCA in a previously healthy elderly male without underlying malignant contribution. EBV testing should be considered in adults presenting with new-onset, transfusion-dependent, normocytic anemia regardless of the presence of malignancy.
